# Une dyspnée révélant une laryngopyocèle

**DOI:** 10.11604/pamj.2018.29.68.14612

**Published:** 2018-01-24

**Authors:** Rachidi Alaoui Siham, Zeriouel Asmae

**Affiliations:** 1 Service de Radiologie, Centre Hospitalier Provincial, Taounate, Maroc

**Keywords:** Laryngocèle, nasofibroscopie laryngée, tomodensitométrie, Laryngocele, laryngeal nasofibroscopy, CT scan

## Abstract

Laryngocele is rare; it is an abnormal dilatation of the laryngeal appendix or of the Morgagni's ventricular pouch. Its size is variable. When it is small, it is usually asymptomatic. When it is large, it can manifest as a cervical anterolateral paralaryngeal mass. Diagnosis is based on CT scan. Treatment approach is still discussed. Endoscopic CO_2_ laser treatment has aroused great interest in recent years. We here report the clinical observation of a 24-year old woman with a 4-year history of intermittent dyspnea. Endoscopic examination showed an increase in the bulge of the right ventricular band associated with arytenoid oedema. Cervical CT scan with contrast medium objectified well-defined, hypodense collection next to the right thyroid cartilage, contrast enhanced in the periphery without bone lysis or lysis of the cartilage. It pressed the vallecula left with discreet infiltration of the surrounding grease. The diagnosis of internal laryngopyocele was suspected, confirming the clinical data. The patient was treated with antibiotics and corticosteroids before cervicotomy since it was not possible to perform endoscopic marsupialisation due to non-availability of the laser.

## Image en médecine

La laryngocèle est une pathologie rare. Elle consiste en une dilatation anormale de l'appendice ou saccule ventriculaire de Morgagni. Sa taille est variable. Petite, elle est généralement asymptomatique. Lorsqu'elle est de grande taille, elle peut se manifester sous la forme d'une masse cervicale, antérolatérale et paralaryngée. L'imagerie reposant sur la tomodensitométrie permet de faire le diagnostic. Sa prise en charge est encore discutée. En effet, le traitement endoscopique au laser CO_2_ a gagné beaucoup d'intérêt ces dernières années. Nous rapportons ici l'observation clinique d'une jeune femme de 24 ans, qui présente depuis quatre ans une dyspnée intermittente L'examen endoscopique montre une augmentation du bombement de la bande ventriculaire droite associée à un œdème de l'aryténoïde. Une TDM cervicale avec injection du produit de contraste a été réalisée et a objectivé une collection hypodense bien limitée en regard du cartilage thyroïde droit prenant le contraste en périphérie sans lyse osseuse ni cartilagineuse. Elle refoule la vallécule à gauche avec une discrète infiltration de la graisse avoisinante. Le diagnostic laryngopyocèle à développement interne est évoqué, confirmant les données de l'examen clinique. Une antibiothérapie et une corticothérapie ont été initiées en attendant la chirurgie par cervicotomie puisque une marsupialisation par voie endoscopique n'est pas possible à cause de non disponibilité du laser.

**Figure 1:**
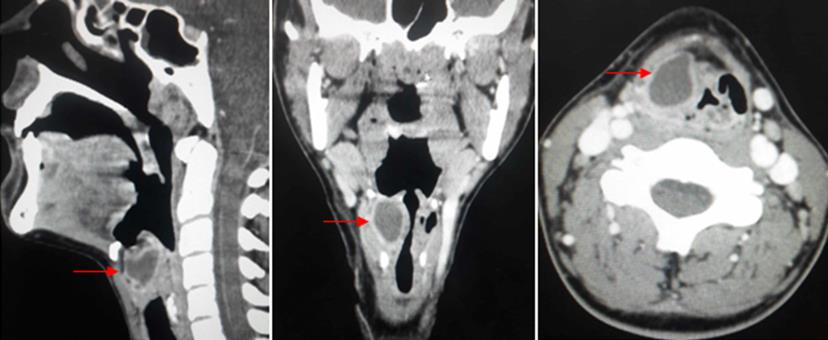
TDM cervicale en coupes: sagitale, coronale et axiale après injection du produit de contratse objectivant une laryngopyocèle interne droite sous forme d’une une collection hypodense bien limitée en regard du cartilage thyroïde droit prenant le contraste en périphérie sans lyse osseuse ni cartilagineuse. Elle refoule la vallécule à gauche avec une discrète infiltration de la graisse avoisinante

